# The PI3-Kinase/mTOR-Targeting Drug NVP-BEZ235 Inhibits Growth and IgE-Dependent Activation of Human Mast Cells and Basophils

**DOI:** 10.1371/journal.pone.0029925

**Published:** 2012-01-27

**Authors:** Katharina Blatt, Harald Herrmann, Irina Mirkina, Emir Hadzijusufovic, Barbara Peter, Sabine Strommer, Gregor Hoermann, Matthias Mayerhofer, Konrad Hoetzenecker, Walter Klepetko, Viviane Ghanim, Katharina Marth, Thorsten Füreder, Volker Wacheck, Rudolf Valenta, Peter Valent

**Affiliations:** 1 Division of Hematology and Hemostaseology, Department of Internal Medicine I, Medical University of Vienna, Vienna, Austria; 2 Ludwig Boltzmann Cluster Oncology, Vienna, Austria; 3 Department for Companion Animals and Horses, Clinic for Internal Medicine and Infectious Diseases, University of Veterinary Medicine Vienna, Vienna, Austria; 4 Department of Clinical Pharmacology, Medical University of Vienna, Vienna, Austria; 5 Department of Laboratory Medicine, Medical University of Vienna, Vienna, Austria; 6 Department of Thoracic Surgery, Medical University of Vienna, Vienna, Austria; 7 Christian Doppler Laboratory for Allergy Research, Division of Immunopathology, Department of Pathophysiology, Center for Pathophysiology, Immunology and Infectiology, Medical University of Vienna, Vienna, Austria; Institut Jacques Monod, France

## Abstract

The phosphoinositide 3-kinase (PI3-kinase) and the mammalian target of rapamycin (mTOR) are two major signaling molecules involved in growth and activation of mast cells (MC) and basophils (BA). We examined the effects of the dual PI3-kinase/mTOR blocker NVP-BEZ235 on growth of normal and neoplastic BA and MC as well as immunoglobulin E (IgE)-dependent cell activation. Growth of MC and BA were determined by measuring ^3^H-thymidine uptake and apoptosis. Cell activation was determined in histamine release experiments and by measuring upregulation of CD63 and CD203c after challenging with IgE plus anti-IgE or allergen. We found that NVP-BEZ235 exerts profound inhibitory effects on growth of primary and cloned neoplastic MC. In the MC leukemia cell line HMC-1, NVP-BEZ235 showed similar IC_50_ values in the HMC-1.1 subclone lacking KIT D816V (0.025 µM) and the HMC-1.2 subclone expressing KIT D816V (0.005 µM). Moreover, NVP-BEZ235 was found to exert strong growth-inhibitory effects on neoplastic MC in a xenotransplant-mouse model employing NMR1-Foxn1^nu^ mice. NVP-BEZ235 also exerted inhibitory effects on cytokine-dependent differentiation of normal BA and MC, but did not induce growth inhibition or apoptosis in mature MC or normal bone marrow cells. Finally, NVP-BEZ235 was found to inhibit IgE-dependent histamine release in BA and MC (IC_50_ 0.5–1 µM) as well as anti-IgE-induced upregulation of CD203c in BA and IgE-dependent upregulation of CD63 in MC. In summary, NVP-BEZ235 produces growth-inhibitory effects in immature neoplastic MC and inhibits IgE-dependent activation of mature BA and MC. Whether these potentially beneficial drug effects have clinical implications is currently under investigation.

## Introduction

Basophils (BA) and mast cells (MC) are effector cells of allergic and other inflammatory reactions [1]–[3]. These cells produce a number of biologically active mediator substances and express receptors for immunoglobulin E (IgE) [1]–[6]. In response to IgE-receptor cross-linking or other stimuli, BA and MC release proinflammatory mediators and thereby contribute to the clinical symptoms in allergic patients [4]–[8]. The capacity of BA and MC to respond to an IgE-dependent trigger (allergen), also termed releasability, depends on genetic factors, signal transduction molecules, the maturation stage of cells, and the presence of triggering cytokines [7]–[9] The severity of an allergic reaction depends on additional factors, including the type of allergen, local organ-specific factors, and the numbers of BA and MC involved in the reaction [10]–[13]. Increased numbers of BA and/or MC are seen in chronic inflammatory disorders, chronic infections, and in certain hematologic disorders [3], [12]–[14]. In systemic mastocytosis (SM), MC numbers are highly elevated in various organs [3], [12]–[14]. In these patients, anaphylactic reactions may be life-threatening and may occur even in the absence of specific (detectable) IgE [12]–[14].

Activation of BA and MC through the IgE receptor is associated with an increase in (activation-linked) cell surface antigens such as CD63, and with activation of downstream signaling pathways [4]–[6], [15]–[19]. Major IgE receptor downstream pathways include the MEK/ERK pathway and the phosphoinositide 3-kinase (PI3-kinase)/Akt pathway [4]–[6], [18], [19]. Especially the latter pathway has been implicated in the process of degranulation and mediator secretion [4]–[6], [18], [19]. In addition, the PI3-kinase is a regulator of growth and survival of MC [20]–[22]. More recently, the PI3-kinase has also been identified as a major signaling molecule responsible for KIT-dependent differentiation and growth of neoplastic MC harboring oncogenic *KIT* mutants [23], [24]. Therefore, PI3-kinase as well as PI3-kinase-downstream signaling molecules such as the mammalian target of rapamycin (mTOR) are considered to represent potential targets of therapy in diseases associated with BA/MC activation or abnormal MC growth [25]–[27]. However, most inhibitory compounds that have been developed in the past cannot be applied in patients because of their unfavorable pharmacological properties and toxicity.

NVP-BEZ235 is a novel orally-bioavailable PI3-kinase inhibitor that has been described to exert growth-inhibitory effects on breast cancer, prostate cancer, and myeloma cell lines [28]–[30]. NVP-BEZ235 inhibits the activtion of all isoforms of the PI3-kinase as well as mTOR [28]. Currently, NVP-BEZ235 is undergoing evaluation in preclinical studies and clinical trials in cancer patients.

In the current study, we examined the effects of NVP-BEZ235 on the growth of normal and neoplastic BA and MC, and on IgE receptor-dependent activation. The results of our studies show that NVP-BEZ235 is a potent inhibitor of growth and activation of human BA and MC. Growth inhibitory effects of the drug were seen not only in normal cells, but also in oncogene-transformed neoplastic BA and MC. These results suggest that NVP-BEZ235 may be an interesting targeted drug for disorders associated with abnormal growth or activation of BA or MC.

## Results

### NVP-BEZ235 inhibits the proliferation of neoplastic BA and MC

**Figure 1:**
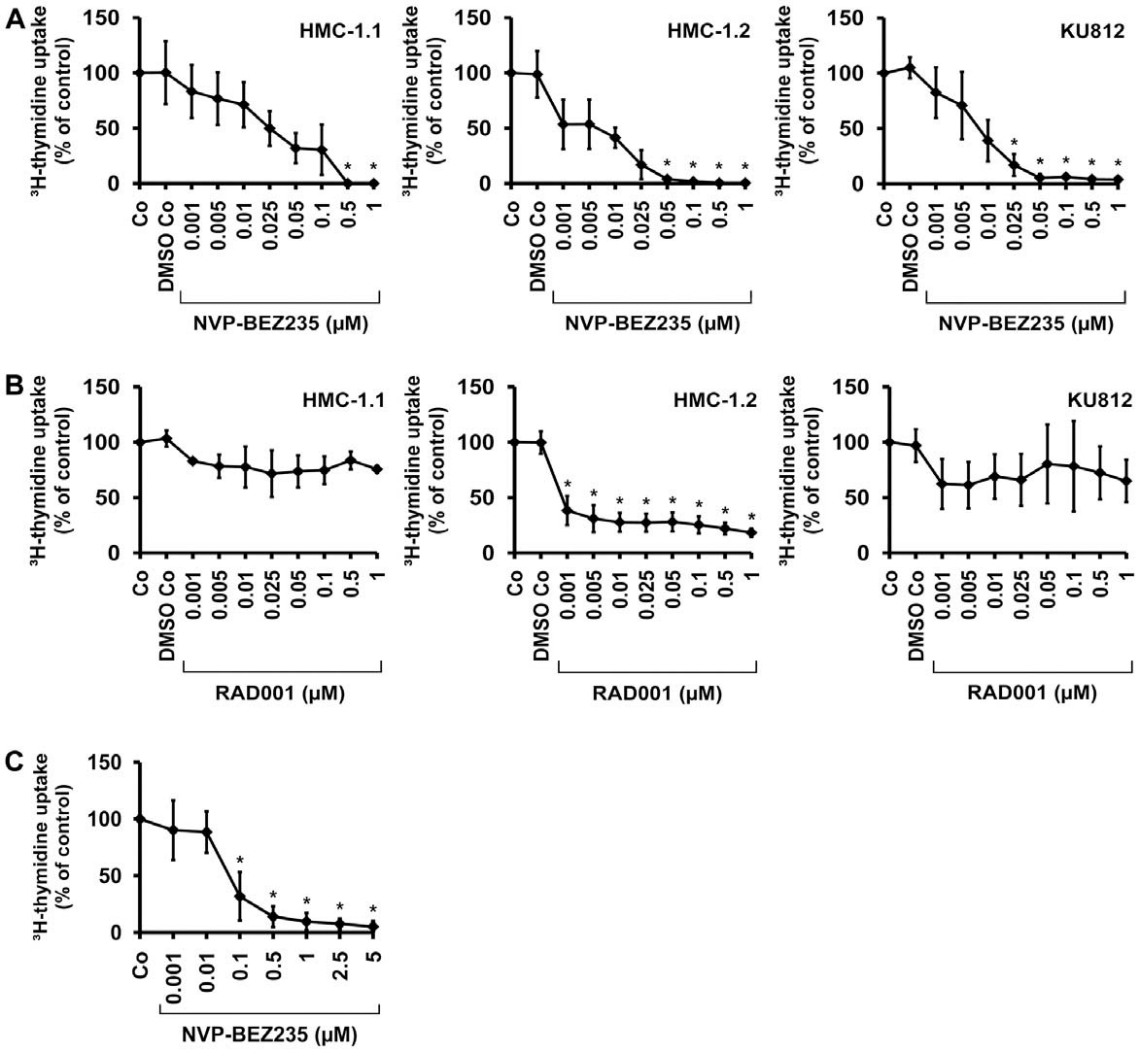
Effects of NVP-BEZ235 on proliferation in HMC-1 cells and KU812 cells. A,B: HMC-1.1 cells (left panels), HMC-1.2 cells (middle panels), and KU812 cells (right panels) were cultured in control medium (Co), control medium with DMSO 1∶1,000 (DMSO Co), or with increasing concentrations (0.001 µM–1 µM) of NVP-BEZ235 (A) or RAD001 (B) at 37°C for 48 hours. Thereafter, ^3^H-thymidine uptake was measured. Results show the percentage of ^3^H-thymidine uptake compared to control (Co) and represent the mean±S.D. of three independent experiments in each cell line. Asterisk (*): p<0.05. C: Bone marrow MNC from six patients with SM were incubated in control medium (Co) or in various concentrations of NVP-BEZ235 (as indicated) at 37°C for 48 hours. Then, ^3^H-thymidine uptake was measured. Results show the percentage of ^3^H-thymidine uptake compared to Co and represent the mean±S.D. of six donors. Asterisk (*): p<0.05.

As determined by ^3^H-thymidine uptake, NVP-BEZ235 was found to inhibit spontaneous proliferation of KU812 cells, HMC-1.1 cells, and HMC-1.2 cells in a dose-dependent manner (Figure 1A). Interestingly, IC_50_ values obtained in HMC-1.1 cells (0.025 µM) were slightly higher when compared to IC_50_ values obtained in HMC-1.2 cells (0.005 µM) (Figure 1A). An unexpected observation was that the mTOR inhibitor RAD001 (everolimus) produced clear growth inhibition in HMC-1.2 cells at low concentrations (IC_50_<0.001 µM), whereas no effects were seen in HMC-1.1 cells or KU812 cells (Figure 1B), suggesting that mTOR may play a particular role in KIT D816V-dependent proliferation. This assumption was confirmed by the fact that wortmannin did not inhibit the proliferation in HMC-1.2 cells over the dose-range tested (0.001–1 µM), whereas LY294002 (known to block mTOR activity at high concentrations) was found to counteract proliferation of HMC-1.2 cells between 5 and 20 µM (Figure S1). We were also able to show that NVP-BEZ235 dose-dependently induces growth arrest in primary neoplastic cells obtained from patients with KIT D816V+ SM (Table 1, Figure 1C). In normal/reactive bone marrow cells (n = 2 donors), no substantial effects of NVP-BEZ235 were seen (Table 1).

**Table 1:** Effects of NVP-BEZ235 on proliferation of normal and neoplastic cells.*

			Age	Serum Tryptase		NVP-BEZ235
Cell Type	Diagnosis**	f/m	years	(ng/ml)	KIT D816V	IC50 (µM)
BM MNC	ISM	f	69	16.8	+	0.1
BM MNC	ISM	m	82	30.7	+	0.1
BM MNC	ISM	f	44	14.0	+	0.1–0.5
BM MNC	ISM	f	39	11.5	+	0.1
BM MNC	SSM	f	56	133.0	+	0.1
BM MNC	ISM	m	66	65.4	+	0.1
BM MNC***	NHL	–	–	n.d.	n.d.	>1
BM MNC***	NHL	–	–	n.d.	n.d.	>1
HMC-1.1	MCL	n.a.	n.a.	n.a.	−	0.025
HMC-1.2	MCL	n.a.	n.a.	n.a.	+	0.005
KU812	CML	n.a.	n.a.	n.a.	−	0.01

*Proliferation was determined by measurement of ^3^H-thymidine uptake as described in the text.

**Diagnoses were established according to published criteria (12,13).

***Normal/reactive BM cells were obtained from patients with NHL without BM involvement.

Abbreviations: f, female; m, male; yrs, years; BM, bone marrow; MNC, mononuclear cells; ISM, indolent systemic mastocytosis; SSM, smoldering systemic mastocytosis; NHL, Non Hodgkin's Lymphoma; MCL, mast cell leukemia; CML, chronic myeloid leukemia; n.d., not determined; n.a., not applicable.

We next examined the effects of NVP-BEZ235 and RAD001 on cell cycle distribution in HMC-1 cells and KU812 cells. As expected, NVP-BEZ235 produced a G1 cell cycle arrest in all three cell lines (Figure S2). RAD001 was also found to induce a G1 cell cycle arrest, but the effect was less pronounced compared to the effect of NVP-BEZ235 (Figure S2). We also examined the effects of LY294002 and wortmannin on cell cycle distribution in HMC-1 cells and KU812 cells. In these experiments, we found that only LY294002, but not wortmannin produces a clear G1 cell cycle arrest in HMC-1 cells and KU812 cells at relatively high concentrations (Figure S2).

### NVP-BEZ235 induces apoptosis in neoplastic BA and MC

**Figure 2:**
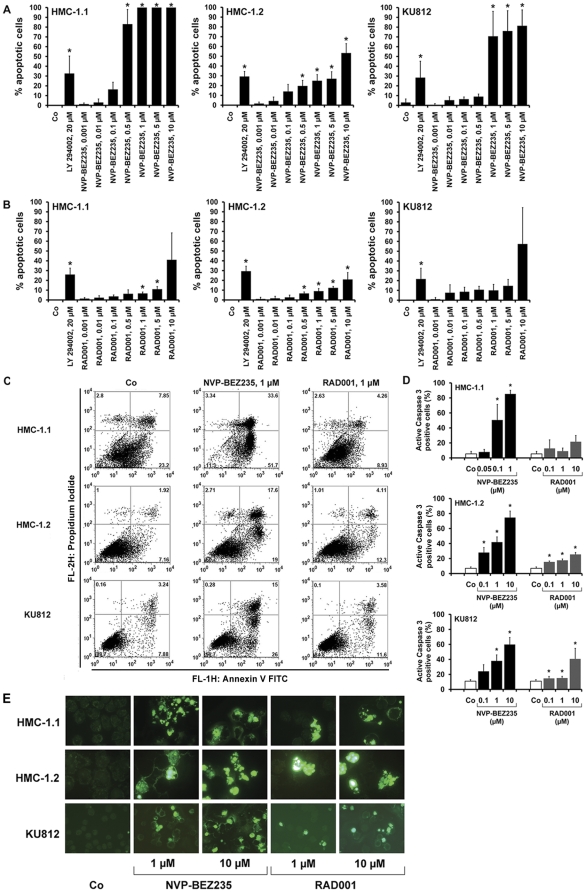
Effects of NVP-BEZ235 on survival of HMC-1 cells and KU812 cells. A,B: HMC-1.1 cells (left panels), HMC-1.2 cells (middle panels), and KU812 cells (right panels) were cultured in control medium (Co), LY294002 (20 µM), or increasing concentrations (0.001 µM–10 µM) of NVP-BEZ235 (A) or RAD001 (B) at 37°C for 48 hours. Then, the numbers (percentage) of apoptotic cells was counted on Giemsa-stained cytospin slides. Results represent the mean±S.D. of three independent experiments. Asterisk (*) indicates p<0.05. C: HMC-1.1 cells (upper panels), HMC-1.2 cells (middle panels) and KU812 cells (lower panels) were incubated in control medium (Co) (left panels) or in medium containing NVP-BEZ235, 1 µM (middle panels) or RAD001, 1 µM (right panel) for 48 hours. After incubation, cells were examined for combined AnnexinV/PI staining by flow cytometry. D: HMC-1.1 (left panel), HMC-1.2 (middle panel), and KU812 cells (right panel) were incubated in control medium (Co) or in various concentrations of NVP-BEZ235 or RAD001 as indicated (37°C, 48 hours). Then, the percentage of active caspase 3-positive cells was determined by flow cytometry. Results represent the mean±S.D. of three independent experiments. Asterisk (*) indicates p<0.05. E: HMC-1.1 cells (upper panels), HMC-1.2 cells (middle panels) and KU812 cells (lower panels) were incubated in control medium (Co) or in medium containing NVP-BEZ235 (1 or 10 µM) or RAD001 (1 or 10 µM) (as indicated) at 37°C for 48 hours. Thereafter, cells were harvested and subjected to Tunel assay and examined by fluorescence microscopy.

To investigate the mechanism of drug-induced growth inhibition, we examined NVP-BEZ235-exposed cells for signs of apoptosis. In these experiments, NVP-BEZ235 was found to induce a dose-dependent increase in apoptotic cells in all three cell lines examined (Figure 2A). An unexpected observation was that apoptosis-inducing effects of NVP-BEZ235 were stronger in HMC-1.1 cells compared to HMC-1.2 cells (Figure 2A). These data suggest that KIT D816V may induce resistance against the apoptosis-inducing effects of NVP-BEZ235, whereas proliferation-inhibitory effects of the drug are not altered by the KIT mutant. We also examined the effects of RAD001 on survival of HMC-1 cells and KU812 cells. However, the drug concentrations required to produce apoptosis were relatively high, and even at the highest concentrations applied (5 and 10 µM), only a small subset of cells were found to be apoptotic (Figure 2B). As a next step, we examined apoptosis-inducing effects of NVP-BEZ235 and RAD001 on KU812 and HMC-1 cells by AnnexinV/propidium iodide (PI) staining (Figure 2C), caspase staining (Figure 2D), and in a Tunel assay (Figure 2E). In all three assays, NVP-BEZ235 was found to induce apoptosis in the three cell lines tested (Figure 2C–2E). As expected, the effects of NVP-BEZ235 on apoptosis-induction were stronger in HMC-1.1 cells compared to HMC-1.2 (Figure 2C–2E). RAD001 also showed some effects in these experiments. However, the effects of RAD001 were far less pronounced compared to the effects seen with NVP-BEZ235 (Figure 2C–2E). In control experiments, NVP-BEZ235 showed no substantial effects on cell viability or the percentage of apoptotic cells (<5% apoptotic cells) in cord blood-derived cultured human MC (data not shown).

### NVP-BEZ235 exerts inhibitory effects on *in vivo* growth of HMC-1.2 cells in a xenotransplantation model

**Figure 3:**
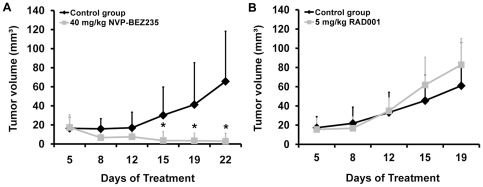
Effect of NVP-BEZ235 on *in vivo* growth of HMC-1.2 cells. Female NMRI-Foxn1^nu^ mice were inoculated with HMC-1.2 cells and were randomized into a treatment group (drug gavaging) and a control group. A: The treatment group (7 mice) received 40 mg/kg NVP-BEZ235 dissolved in NMP and PEG300 *per os* every day, and the control group (6 mice) received NMP and PEG300 alone for 22 days. B: The treatment group (5 mice) received 5 mg/kg RAD001 dissolved in distilled water *per os*, and the control group (5 mice) received sodium chloride *per os* for 19 days. Tumor size is shown in the vertical axis and is expressed in mm^3^. Results represent the mean±S.D. in each group. Asterisk (*) indicates p<0.05 compared to the control group.

To confirm the inhibitory effects of NVP-BEZ235 on growth of HMC-1 cells, we examined the drug in a xenotransplant model using HMC-1.2 cells and NMR1-Foxn1^nu^ mice. In these mice, HMC-1 cells formed tumor nodules after subcutaneous injection within 1–2 weeks. Administration of NVP-BEZ235 (40 mg/kg, *per os* daily from day 5) resulted in a profound inhibition of tumor formation in all mice examined (Figure 3A). By contrast, no inhibitory effects of RAD001 on *in vivo* growth of HMC-1 cells in nude mice could be substantiated (Figure 3B). All in all, these data suggest that NVP-BEZ235 exerts anti-neoplastic effects on growth of malignant MC *in vivo*.

### NVP-BEZ235 inhibits cytokine-dependent differentiation of human BA and MC

**Figure 4:**
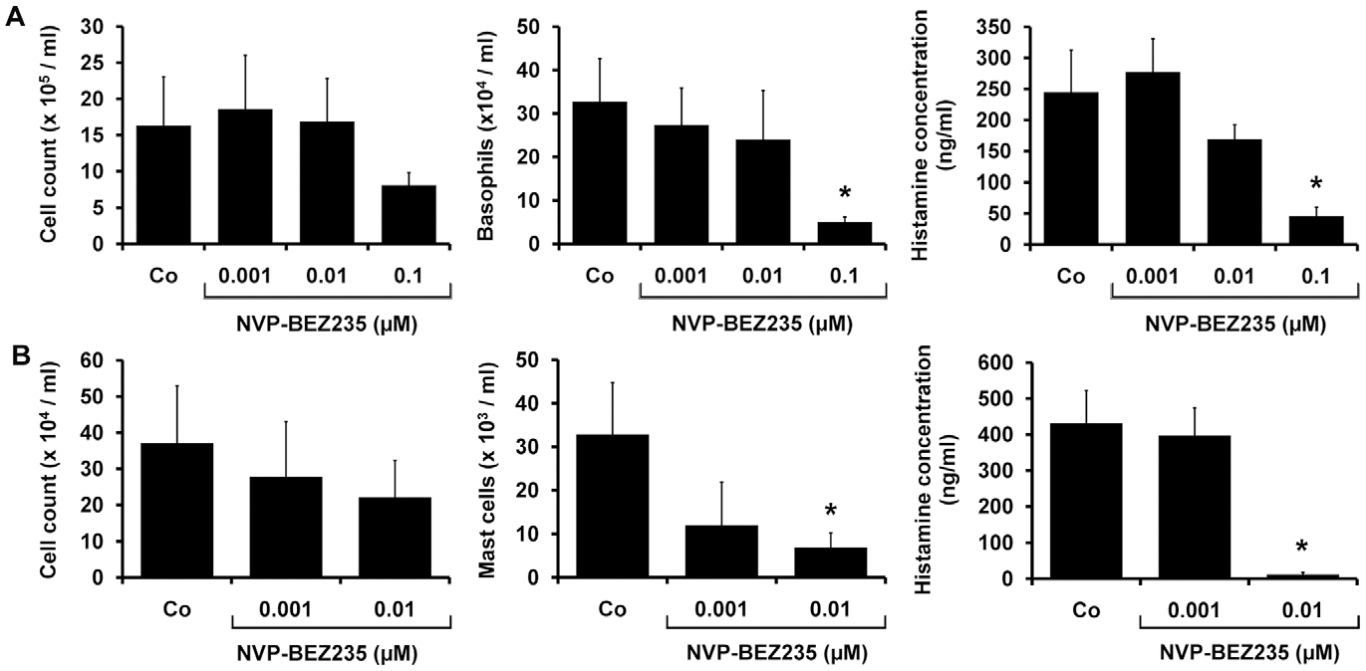
Effects of NVP-BEZ235 on cytokine-dependent differentiation of human BA and MC. A: Isolated cord blood MNC (0.5×10^6^/ml) were cultured in 6-well plates (Costar, Cambridge, MA) in RPMI medium containing 10% FCS and IL-3 (100 ng/ml) with or without NVP-BEZ235 (0.001–0.1 µM) at 37°C for two weeks. Thereafter, the total cell numbers per well (left panel), the total numbers of BA per well determined from total cell counts and differential counts obtained in Giemsa-stained slides (middle panel), and the total cellular histamine levels per well assessed by radioimmunoassay (right panel), were determined. B: Cord blood MNC were cultured in RPMI medium containing 10% FCS, SCF (100 ng/ml), and IL-6 (100 ng/ml), with or without NVP-BEZ235 (0.001–0.01 µM) at 37°C for four weeks. Thereafter, the total cell numbers per well (left panel), the total numbers of MC per well determined from total cell counts and differential counts (middle panel), and the total cellular histamine levels per well (right panel), were determined. Results represent the mean±S.D of three independent experiments. Asterix (*): p<0.05.

A generally accepted hypothesis is, that cytokine-dependent growth of normal myeloid cells is dependent on PI3-kinase activity [20]–[23]. We therefore asked whether NVP-BEZ235 would also inhibit cytokine-dependent differentiation of normal BA and MC from their immature cord blood-derived progenitors. In these experiments, NVP-BEZ235 was found to suppress IL-3-dependent differentiation of BA as well as SCF-dependent differentiation of MC in a dose-dependent manner (Figure 4). In particular, whereas, NVP-BEZ235 exerted only mild effects on the total number of cultured cells, a significant inhibitory effect on cytokine-dependent development of BA and MC was seen. IC_50_ values obtained for NVP-BEZ235 in these experiments were by far lower (<0.1 µM) compared to IC_50_ values obtained in cell activation experiments.

### Effects of NVP-BEZ235 on expression of phosphorylated signaling molecules in neoplastic BA (KU812) and MC (HMC-1)

**Figure 5:**
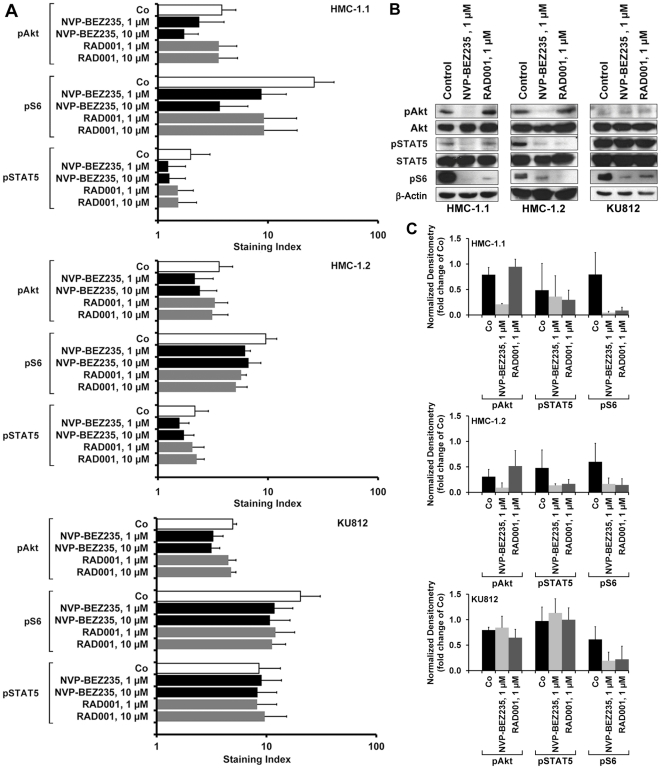
Effects of NVP-BEZ235 on expression of pAkt, pS6, and pSTAT5 in HMC-1 cells and KU812 cells. A: HMC-1.1 cells (upper panel), HMC-1.2 cells (middle panel), and KU812 cells (lower panel) were incubated with control medium (Co) or medium containing NVP-BEZ235 (1 or 10 µM) or RAD001 (1 or 10 µM) at 37°C for 4 hours. Thereafter, cells were permeabilized and stained with antibodies against pAkt (S473), pS6, or pSTAT5 as described in the text. Expression of phosphorylated (p) signaling molecules in HMC-1 cells and KU812 cells were determined by flow cytometry. Results show the staining index (mean fluorescence intensity values corrected for the isotype) and represent the mean±S.D. from three independent experiments. B: HMC-1.1 cells (upper panel), HMC-1.2 cells (middle panel), and KU812 cells (lower panel) were cultured in the absence or presence of NVP-BEZ235 (1 µM) RAD001 (1 µM) at 37°C for 4 hours. Thereafter, cells were lysed and Western blotting was performed using antibodies against pAkt (S473), Akt, pSTAT5, STAT5, pS6 and β-Actin. C: Western blot results were quantified by densitometry in HMC-1.1 cells (upper panel), HMC-1.2 cells (middle panel), and KU812 cells (lower panel). Results represent the mean±S.D. from three independent experiments.

**Figure 6:**
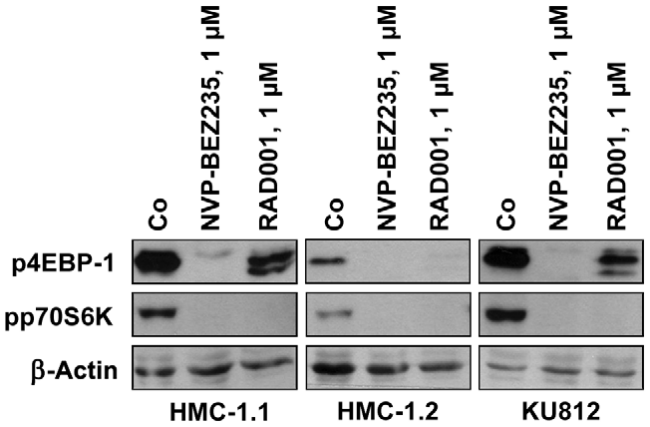
Effects of NVP-BEZ235 on expression of p4EBP-1 and pp70S6K in HMC-1 cells and KU812 cells. HMC-1.1 cells (left panel), HMC-1.2 cells (middle panel), and KU812 cells (right panel) were cultured in the absence or presence of NVP-BEZ235 (1 µM) or RAD001 (1 µM) at 37°C for 4 hours. Thereafter, cells were lysed, and Western blotting was performed using antibodies against p4EBP-1, pp70S6K, and β-Actin.

To confirm that NVP-BEZ235 exerts effects on HMC-1 cells and KU812 cells through specific signal transduction molecules, we performed flow cytometry experiments using antibodies against phosphorylated Akt (pAkt, S473), pS6, and pSTAT5. We found that NVP-BEZ235 substantially downregulated activation of Akt and S6 in HMC-1.1 cells, and less effectively in HMC-1.2 and KU812 cells (Figure 5A). As expected, RAD001 was found to counteract expression of pS6 in HMC-1 cells and KU812 cells, but did not inhibit pAkt (S473) (Figure 5A). Interestingly, NVP-BEZ235 and RAD001 slightly decreased the expression of pSTAT5 in HMC-1.1 cells and HMC-1.2 cells (Figure 5A). By contrast, no effect of NVP-BEZ235 on pSTAT5 expression in KU812 cells was seen. Data obtained by flow cytometry were confirmed by Western blot experiments (Figure 5B and 5C). Again, NVP-BEZ235 was found to downregulate expression of pAkt (S473), pSTAT5, and pS6 in HMC-1.1 cells and HMC-1.2 cells, and expression of pAkt and pS6 in KU812 cells (Figure 5B and 5C). Similar to our flow cytometry data, NVP-BEZ235 did not downregulate activation of pSTAT5 in KU812 cells. As determined by densitometry, RAD001 was found to slightly upregulate expression of pAkt (Figure 5C). NVP-BEZ235 and RAD001 were found to downregulate expression of pp70S6K and p4EBP-1 in HMC-1 cells and KU812 cells (Figure 6). However, whereas NVP-BEZ235 was found to exert strong inhibitory effects on pAkt, pp70S6K, and p4EBP-1, RAD001, which is a selective mTORC1 blocker, was found to strongly inhibit expression of pp70S6K and p4EBP-1 (Figure 6), but did not inhibit expression of pAkt (Figure 5). Finally, we explored whether NVP-BEZ235 or RAD001 would interfere with pERK expression in HMC-1 cells or KU812 cells. However, under the experimental conditions applied, neither NVP-BEZ235 nor RAD001 were found to counteract phosphorylation of ERK in HMC-1 cells and KU812 cells (Figure S3).

### NVP-BEZ235 inhibits IgE-dependent mediator release in human MC

**Figure 7:**
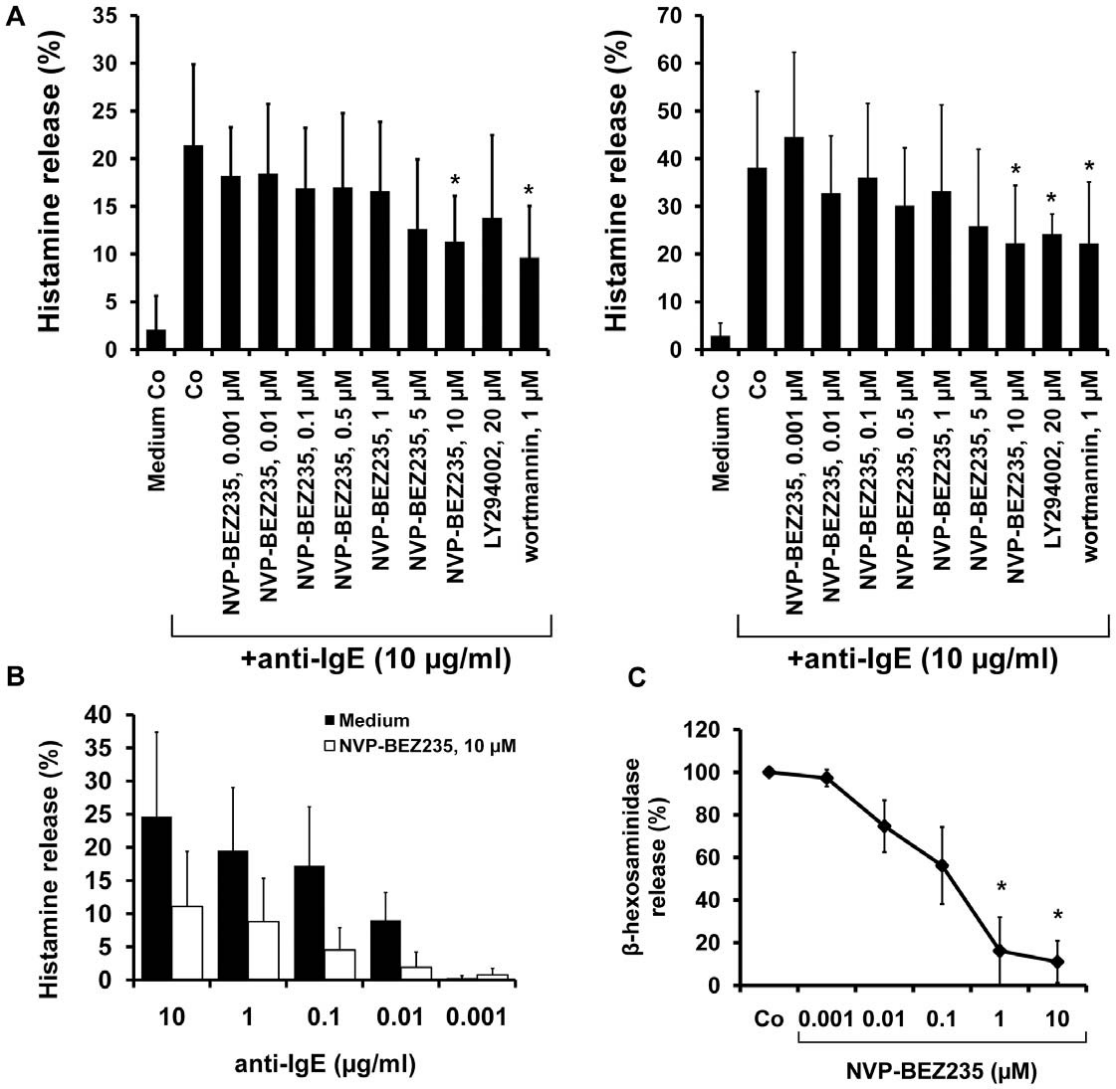
Effects of NVP-BEZ235 on mediator secretion in human MC. A: IgE-preloaded cultured cord blood-derived MC (left panel) and IgE-preloaded lung MC (right panel) were incubated in control medium, various concentrations of NVP-BEZ235 (as indicated), LY294002 (20 µM), or wortmannin (1 µM) (30 minutes, 37°C). Then, cells were challenged with anti-IgE (10 µg/ml) at 37°C for 30 minutes. After incubation, histamine release was determined. Results show the percentage of released histamine and represent the mean±S.D. of four (cultured MC, left panel) or five independent experiments (5 lung MC samples, right panel). Asterisk (*): p<0.05. B: Cultured MC were incubated in the presence of control medium (indicated by black bars) or medium containing NVP-BEZ235, 10 µM (open bars) for 30 minutes at 37°C. Then, cells were incubated with various concentrations of anti-IgE as indicated. After incubation, supernatants and lysates were harvested and examined for histamine content. Histamine release was calculated as percent of total histamine. Results show the percent of released histamine and are expressed as mean±S.D. of three independent experiments. Asterisk (*): p<0.05. C: MC preloaded with NIP(5)-BSA-specific IgE were cultured in control medium (Co) or in various concentrations of NVP-BEZ235 (0.001–10 µM) for 30 minutes at 37°C. Thereafter, cells were washed and incubated with NIP(5)-BSA (100 ng/ml) for 90 minutes (37°C). Then, cell-free supernatants were collected and β-hexosaminidase release was measured. Results are expressed in percent of response induced by anti-IgE without NVP-BEZ235 and represent the mean±S.D of three independent experiments. Asterisk (*) indicates p<0.05.

NVP-BEZ235 was found to counteract IgE-dependent release of histamine in cultured cord blood progenitor-derived MC as well as in mature human lung MC (Figure 7A). The effects of NVP-BEZ235 on histamine release were mild, but dose-dependent and seen at all concentrations of anti-IgE applied (Figure 7B). In addition, NVP-BEZ235 was also found to inhibit the IgE-dependent release of β-hexosaminidase in MC (Figure 7C). However, compared to BA, higher concentrations of NVP-BEZ235 (1–10 µM) were required to block mediator secretion in MC. RAD001 showed no effects on mediator release in MC (not shown).

### NVP-BEZ235 inhibits IgE-dependent histamine release in human BA

**Figure 8:**
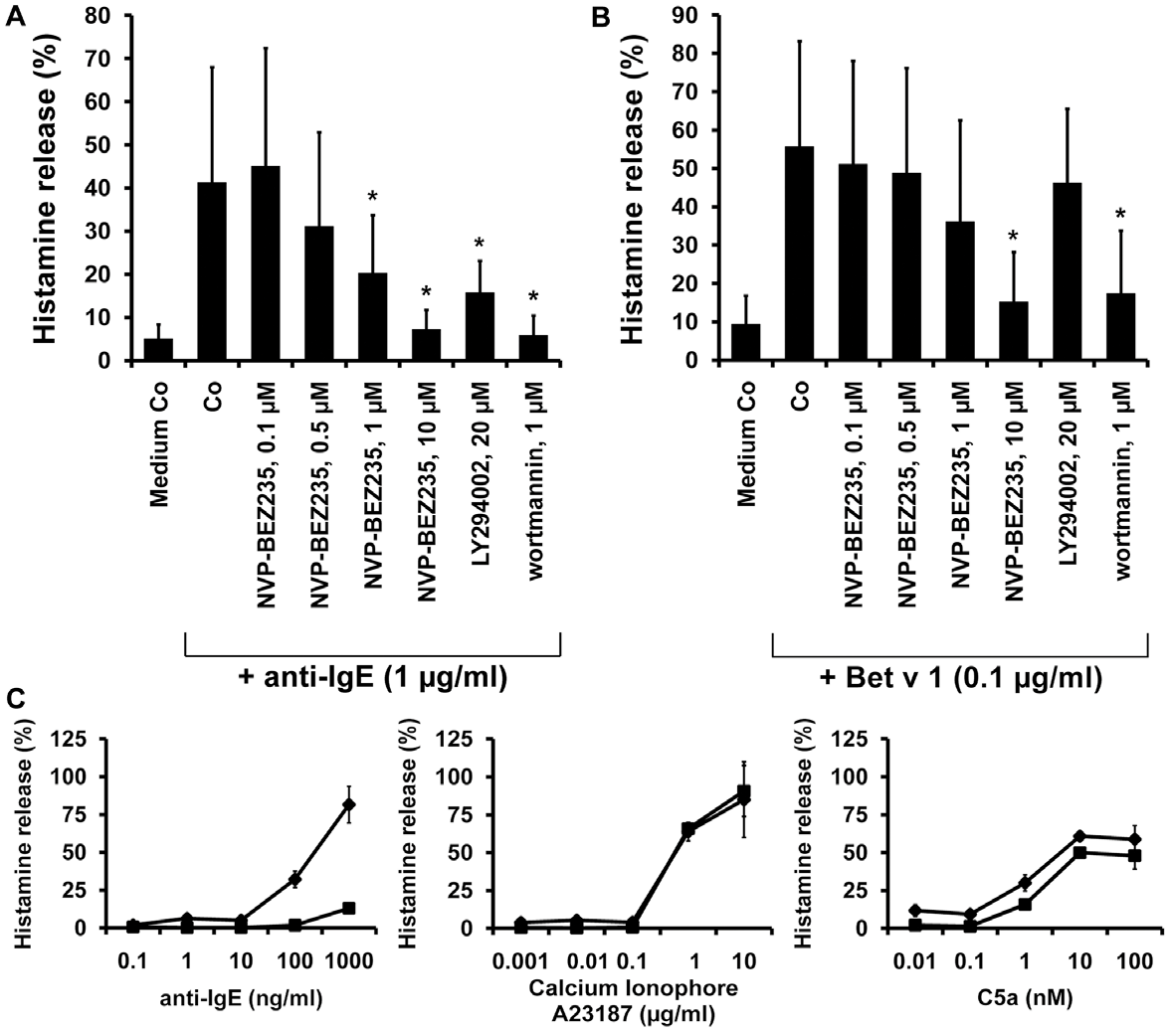
Effects of NVP-BEZ235 on IgE-mediated histamine release in human BA. BA obtained from healthy donors (A) or patients allergic to Bet v 1 (B) were preincubated with control medium (Medium Co), various concentrations of NVP-BEZ235 (as indicated), LY294002 (20 µM), or wortmannin (1 µM) at 37°C for 30 minutes. Then, cells were exposed to anti-IgE (1 µg/ml; healthy controls) or recombinant allergen (0.1 µg/ml of Bet v 1; allergic patients) at 37°C for 30 minutes. After centrifugation, histamine concentrations were determined in supernatants and cell-lysates by RIA. Histamine release is expressed as percentage of total histamine. Results represent the mean±S.D. from five donors. Asterisk (*) indicates p<0.05. C: BA obtained from one healthy donor were incubated in control medium (♦-♦) or medium containing NVP-BEZ235, 10 µM (▪-▪) for 30 minutes at 37°C. Then, cells were incubated with various concentrations (as indicated) of anti-IgE (left panel), Ca-ionophore A23187 (middle panel), or recombinant C5a (right panel) for another 30 minutes (37°C). After incubation, supernatants and lysates were harvested and examined for their histamine content. Histamine release was calculated as percent of total histamine. Data are expressed as mean±S.D. of triplicates.

At 1 µM and 10 µM, NVP-BEZ235 was found to inhibit anti-IgE-induced release of histamine from normal BA in all donors (n = 5) examined (Figure 8A). The effects of NVP-BEZ235 were dose-dependent with IC_50_ values ranging between 0.5 and 1 µM. NVP-BEZ235 was also found to counteract allergen-induced histamine secretion in BA obtained from allergic patients (Figure 8B). Again the effects of NVP-BEZ235 on allergen-induced histamine release in BA were dose-dependent. To define whether the effects of NVP-BEZ235 on histamine release were mediated through mTOR, we applied the mTOR-specific drug RAD001. However, in contrast to NVP-BEZ235, RAD001 showed no significant effects on IgE-mediated histamine release (not shown). NVP-BEZ235 failed to inhibit the A23187-induced or C5a-induced release of histamine in human BA (Figure 8C). These data suggest that NVP-BEZ235 blocks IgE-mediated histamine release through inhibition of PI3-kinase activity in human BA, a conclusion that was supported by the observation that wortmannin and LY294002 also blocked IgE-dependent histamine release in normal BA (Figure 8A and 8B). The effects of wortmannin and LY294002 on histamine release in normal BA were dose-dependent (Figure S4).

### NVP-BEZ235 inhibits the IgE-dependent upregulation of activation-linked cell surface antigens on BA and MC

**Figure 9:**
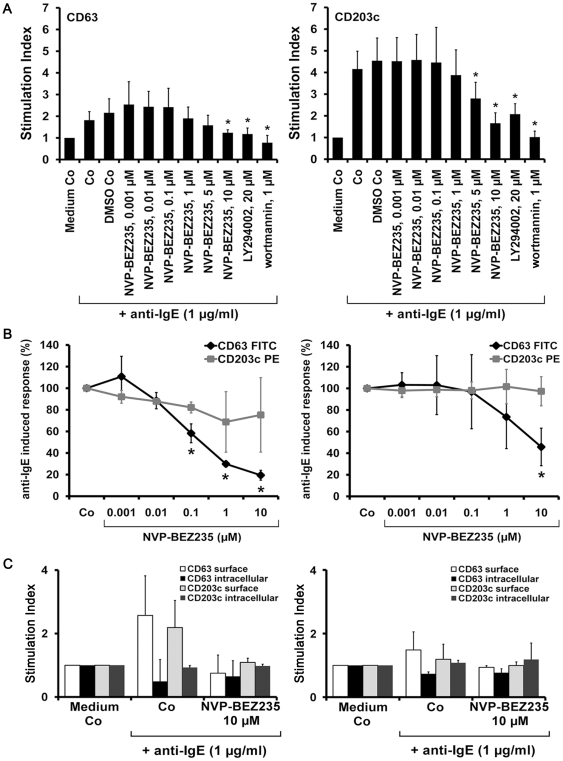
Effects of NVP-BEZ235 on expression of activation-linked cell surface antigens on BA and MC. A: BA in whole blood samples were preincubated in control medium (Medium Co) or in medium containing various concentrations of NVP-BEZ235 (0.001–10 µM), LY294002 (20 µM), or wortmannin (1 µM) at 37°C for 15 minutes. Then, cells were exposed to anti-IgE antibody E-124.2.8 (1 µg/ml) for another 15 minutes (37°C). Thereafter, cells were stained with mAb directed against CD63 (left panel) or CD203c (right panel), and analyzed by multicolor flow cytometry as described in the text. BA were defined as CD203c-positive cells in all samples. Anti-IgE-induced upregulation of CD antigens was calculated from mean fluorescence intensities (MFIs) obtained with stimulated (MFIstim) and unstimulated (MFIcontrol) cells and was expressed as SI (MFIstim∶MFIcontrol). Results show SI values and represent the mean±S.D. from five normal donors (the same as shown in Figure 1A). Asterisk (*) indicates p<0.05. B: IgE-preloaded cultured MC (left panel) or IgE-preloaded lung MC (right panel) were incubated in control medium (Co) or in various concentrations of NVP-BEZ235 (as indicated) at 37°C for 40 minutes. Then, cells were incubated with anti-IgE antibody E-124.2.8 (10 µg/ml) for 30 minutes (37°C). After incubation, expression of CD63 (♦-♦) and CD203c (□-□) on MC was analyzed by flow cytometry. Results show the percent-inhibition of anti-IgE-induced upregulation of CD63 or CD203c on MC, and represent the mean±S.D. from three (cultured MC) or five (lung MC) independent experiments. Asterisk (*): p<0.05. C: Ficoll-isolated BA (left panel) and lung MC (right panel) were preincubated in control medium (Co) or with 10 µM NVP-BEZ235 at 37°C for 15 minutes. Then, cells were exposed to control medium or anti-IgE antibody E-124.2.8 (1 µg/ml) for another 15 minutes (37°C). Thereafter, cells were stained with mAb directed against CD63 or CD203c and analyzed by flow cytometry using a multicolor surface staining protocol and a cytoplasmic staining protocol. Results show SI values of surface CD63 (white bars), cytoplasmic CD63 (black bars), surface CD203c (light grey bars) and cytoplasmic CD203c (dark grey bars) and represent the mean±S.D. from three independent experiments.

IgE receptor-dependent activation of BA and MC is accompanied by an increased expression of activation-linked cell surface antigens [15]–[17], [31]. In the present study, we found that NVP-BEZ235 inhibits anti-IgE-induced upregulation of CD63 and CD203c on BA in a dose-dependent manner (IC_50_: 5–10 µM) (Figure 9A). Wortmannin and LY294002 were also found to inhibit anti-IgE-induced upregulation of CD63 and CD203c (Figure 9A; Figure S5A). In cultured human MC and primary lung MC, NVP-BEZ235 was found to inhibit the IgE-dependent upregulation of CD63, whereas no substantial effects on IgE-dependent upregulation of CD203c were seen (Figure 9B). To study the mechanism of anti-IgE-induced upregulation of CD63 and CD203c, we performed cell surface and cytoplasmic staining experiments with IgE receptor cross-linked BA and MC. In these experiments, we found that IgE receptor cross-linking is followed by a rapid increase in surface expression of CD63 and CD203c on BA, whereas the intracellular levels of CD63 and CD203c slightly decreased (Figure 9C, left panel). In lung MC, the same phenomenon was observed for CD63. Both the anti-IgE-induced upregulation of CD63 on the surface, and the decrease in cytoplasmic CD63 were reverted by NVP-BEZ235 (Figure 9C, right panel). In a next step, we examined the effects of NVP-BEZ235 on expression of CD63 and CD203c on KU812 and HMC-1 cells. We found that NVP-BEZ235 downregulated the expression of CD63 in KU812 cells in a dose-dependent manner (Figure S5B). By contrast, no effects of NVP-BEZ235 on expression of CD63 in HMC-1 cells were found, and we were also unable to demonstrate substantial effects of NVP-BEZ235 on expression of CD203c on KU812 or HMC-1 cells (not shown). Similarly, the mTOR inhibitor RAD001 and the other PI3-kinase blockers applied (LY294002, wortmannin) did not inhibit the expression of CD63 or CD203c on KU812 cells or HMC-1 cells. These data suggest that expression of CD63 and CD203c on neoplastic BA and MC is not dependent on PI3-kinase activation.

## Discussion

Growth, survival, and IgE-dependent activation of BA and MC are regulated by a complex network of signal transduction cascades and molecules, including various protein kinases [3]–[5], [21]–[24]. These signaling molecules are considered to represent potential therapeutic targets in allergic patients as well as in patients with MC proliferative disorders where abnormal MC growth and activation represent major clinical problems [24]–[27], [32]. Recent data suggest that the PI3-kinase is a key regulator of growth and activation of human BA and MC [20]–[24]. We here show that the PI3-kinase/mTOR-targeting drug NVP-BEZ235 exerts major growth-inhibitory effects on neoplastic BA and MC, and inhibits IgE-dependent mediator secretion.

Initially, NVP-BEZ235 was developed as a dual inhibitor of PI3-kinase and mTOR [28]. In the present study, we were able to confirm the PI3-kinase-blocking and mTOR-targeting activity of the drug. In particular, as assessed by Western blotting and flow cytometry, NVP-BEZ235 was found to downregulate the expression of pAkt (S473) and pS6 in HMC-1 cells, whereas the mTOR-targeting drug RAD001 only blocked pS6 activity. However, depending on the cell type and function analyzed (growth or activation), PI3-kinase and mTOR may play a quite different role. In fact, BA- and MC-activation could only be downregulated by PI3-kinase inhibitors but not by the mTOR blocker RAD001. On the other hand, mTOR seems to play a particular role in neoplastic growth of MC carrying the KIT mutant D816V.

Interestingly, RAD001 did not mimic most of the effects of NVP-BEZ235 on mediator secretion in BA or MC, and where effects of RAD001 were seen, they were less pronounced or neglectible compared to effects seen with NVP-BEZ235. These data suggest that the inhibitory effects of NVP-BEZ235 on BA and MC are primarily mediated via PI3-kinase inhibition rather than mTOR inhibition, and that other PI3-kinase-downstream signaling molecules may be involved in the effects of NVP-BEZ235 on activation of BA and MC. The identity of these downstream molecules remains at present unknown. Another explanation would be that RAD001 is a selective mTORC1 inhibitor that is known to even activate Akt in a negative feed-back loop. Accordingly, we found that RAD001 slightly upregulates pAkt expression in HMC-1 cells. An alternative explanation for the differential effects of RAD001 and NVP-BEZ235 would be that NVP-BEZ235 acts on BA and MC via PI3-kinase/mTOR-independent targets. Indeed, in KU812 cells, NVP-BEZ235 showed only slight effects on PI3-kinase phosphorylation, although the drug exerted strong growth-inhibitory effects. On the other hand, the target spectrum of NVP-BEZ235 has been well-defined and no other major kinase targets have been identified so far [28]. Finally, most effects of NVP-BEZ235 on normal BA were copied by the PI3-kinase inhibitors wortmannin and LY294002. An interesting exception and observation was that LY294002 did not inhibit IgE-dependent histamine release in BA in allergic patients, suggesting that BA in allergic patients may be resistant against release-inhibitory effects of this agent.

An unexpected result was that NVP-BEZ235 not only downregulates expression of pAkt (S473) and pS6 in HMC-1 cells, but also expression of pSTAT5. This is of particular interest for several reasons. First, STAT5 has been implicated as a major regulator of growth, survival, and activation of MC [24], [33]–[35]. Second, it has been described that in neoplastic MC, STAT5 forms a signaling complex with PI3-kinase that can be triggered/activated by oncogenic KIT mutants [24]. Based upon these observations, it is tempting to speculate that NVP-BEZ235 acts inhibitory on this PI3-kinase/STAT5 signaling complex in KIT D816V+ neoplastic MC. The possibility that NVP-BEZ235 directly binds to STAT5 seems rather unlikely.

Although NVP-BEZ235 was found to inhibit both the proliferation and activation of human BA and MC in this study, some interesting differences concerning drug actions were observed. The most apparent and probably relevant difference was the higher IC_50_ values required to block cell activation and mediator secretion compared to doses required for growth-inhibition. The biochemical basis of this difference remains unknown. Possible explanations would be different targets involved in the reactions or the different time of incubation used to treat cells. Clinically, the lower IC_50_ values would argue for the use of NVP-BEZ235 to counteract growth of neoplastic BA or MC rather than to block BA- or MC-activation in allergic disorders.

We were also able to confirm the major growth-inhibitory effects of NVP-BEZ235 on neoplastic MC in a xenotransplant mouse model using HMC-1.2 cells. Again NVP-BEZ235 was found to block the growth of HMC-1.2 in this model, whereas RAD001 showed no substantial effects. This discrepancy may be due to the lower dose of RAD001 applied (5 mg/kg/day) compared to the dose of NVP-BEZ235 (40 mg/kg/day). However, we were unable to increase the dose of RAD001 because of its known toxicity. The strong inhibitory effects of NVP-BEZ235 on *in vivo* growth of HMC-1 cells may be of clinical significance.

An interesting observation was that NVP-BEZ235 produces differential effects on survival in HMC-1.1 cells lacking KIT D816V and HMC-1.2 cells expressing KIT D816V. In particular, although at high concentrations, the drug produced massive apoptosis in HMC-1.1 cells (>90% apoptotic cells), only a small subset of HMC-1.2 cells underwent apoptosis regardless of the drug concentrations applied. This was an unexpected outcome as the effects of NVP-BEZ235 on proliferation of the two HMC-1 subclones were comparable. One possible explanation for these results would be that the *KIT* mutation D816V confers specific resistance against apoptosis-inducing effects of NVP-BEZ235. In this regard it is noteworthy that this mutant also causes resistance against established KIT kinase inhibitors including imatinib [36], [37]. An alternative explanation would be that the effects of NVP-BEZ235 on growth of HMC-1.2 cells were primarily mediated through inhibition of the cell cycle-regulator mTOR rather than PI3-kinase inhibition. This assumption would be supported by the observation that the mTOR blocker RAD001 produced marked growth inhibition in HMC-1.2 cells expressing KIT D816V, but not in HMC-1.1 cells lacking KIT D816V, which confirmed previous data obtained with the mTOR-blocker rapamycin [38], [39]. Moreover, the PI3-kinase blocker LY294002 only produced growth inhibitory effects in HMC-1.2 cells when applied at high concentrations known to block mTOR activity, whereas no effects were seen with wortmannin. All in all, these data suggest that KIT D816V-driven proliferation of neoplastic MC may be particularly sensitive against mTOR-targeting drugs.

Since mTOR is a major regulator of cell cycle progression we were also interested to learn whether NVP-BEZ235 would interfere with cell cycle progression in HMC-1 cells and KU812 cells. Indeed, NVP-BEZ235 was found to induce a G1 cell cycle arrest in HMC-1.1, HMC-1.2, and KU812 cells. RAD001 also induced a G1 cell cycle arrest although the effect of RAD001 was less pronounced compared to NVP-BEZ235. Finally, LY294002 also produced a G1 cell cycle arrest in these cells at high concentrations, whereas wortmannin showed no effects. These results were somehow unexpected as RAD001 and LY294002 did not produce growth inhibition in HMC-1.1 cells, and RAD001 also failed to inhibit the proliferation of KU812 cells, whereas NVP-BEZ235 exerted effects on growth in all cell lines. This discrepancy may be explained by the fact that not only mTOR but also other (PI3-kinase-downstream) cell cycle regulators play a role in the proliferation of these cells. Alternatively, HMC-1.1 cells are resistant against RAD001, LY294002, and wortmannin independent of mTOR and PI3-kinase-activity.

Several cell surface molecules are typically upregulated on BA and MC after cross-linking of IgE receptors [15]–[17], [40]. In addition, these activation-linked surface antigens are often overexpressed on neoplastic MC when compared to normal cells [13], [14], [40]. We asked whether NVP-BEZ235 would interfere with IgE-mediated expression of CD63 and CD203c on BA and MC. In these experiments, we found that NVP-BEZ235 inhibits the IgE-dependent upregulation of CD203c on BA and IgE-dependent upregulation of CD63 on MC. By contrast, no drug effect on IgE-dependent expression of CD63 on BA or IgE-mediated expression of CD203c on MC was seen. These observations suggest that NVP-BEZ235 may be insufficient to block all relevant pathways in activated BA or MC. Alternatively, expression of CD63 in BA and of CD203c on MC is regulated by PI3-kinase-independent mechanisms. In this regard it is noteworthy, that NVP-BEZ235 did not counteract expression of CD63 or CD203c on HMC-1 cells, and in KU812 cells only a slight effect on CD63 expression was seen.

Based on the intriguing effects of NVP-BEZ235 on growth (survival) and activation of BA and MC, one could speculate on clinical applications and possible indications in future trials. Likewise, in advanced SM, patients suffer not only from the aggressive and sometimes devastating infiltration of MC, but also from mediator-related symptoms [11]–[13]. For these patients, a drug that would block both MC growth and MC activation would be of interest. Our *in vitro* data and data obtained in a mouse xenotransplant model would be in favor of such a new treatment concept. However, so far no clinical studies using NVP-BEZ235 in allergic patients or patients with mastocytosis have been conducted. In summary, our data show that NVP-BEZ235 inhibits growth and activation of human BA and MC. Further preclinical studies are required to confirm this drug effect and to explore whether the drug is a candidate to be tested in patients with MC disorders or MC/BA activation.

## Materials and Methods

### Ethics Statement

All studies were approved by the institutional review board of the Medical University of Vienna and conducted in accordance with the declaration of Helsinki. Written informed consent was obtained from patients or mothers (cord blood) in each case.

Animal studies were approved by the ethics committee of the Medical University of Vienna (3544/115-10/08), and carried out in accordance with guidelines for animal care and protection and protocols approved by the Austrian law and the ethics committee of the Medical University of Vienna (GZ 66.009/0322-II/10b/2008).

### Reagents

NVP-BEZ235 [28] and RAD001 (everolimus) were kindly provided by Novartis Pharma AG, Basel, Switzerland. Stock solutions of drugs were prepared by dissolving in dimethylsulfoxide (DMSO) (Merck, Darmstadt, Germany). Histamine release buffer (HRB), anti-IgE antibody E-124.2.8, and a histamine radioimmunoassay (RIA) were purchased from Immunotech (Marseille, France). RPMI 1640 medium, Iscove's modified Dulbecco's medium (IMDM), and fetal calf serum (FCS) were from PAA laboratories (Pasching, Austria), Ca-ionophore A23187 and recombinant C5a from Sigma Aldrich (St. Louis, MO), LY294002 and wortmannin from Calbiochem (San Diego, CA), and L-glutamine from Gibco Life Technologies (Gaithersburg, MD). Recombinant human (rh) interleukin (IL)-3, IL-4, IL-6, and stem cell factor (SCF) were from Novartis Pharma AG. NIP(5)-BSA was purchased from Biosearch Technologies (Novato, CA), a human NIP-specific IgE (clone Jw8.1) from AbD Serotec (Düsseldorf, Germany), collagenase type II from Worthington (Lakewood, NJ), Stem Span serum-free medium (SFM) from Stem Cell Technologies (CellSystems, St Katharinen, Germany), and the CD133 Microbead Kit from Miltenyi Biotec (Bergisch Gladbach, Germany). The FITC-labeled CD63 antibody CLB-gran12 and PE-labeled CD203c antibody 97A6 were purchased from Beckman Coulter (Fullerton, CA), the unlabeled monoclonal antibody (mAb) CLB-gran12 from Sanquin Reagents (Amsterdam, Netherlands), and a PE-labeled and unlabeled CD203c mAb (NP4D6) from Biolegend (San Diego, CA).

### Isolation and culture of blood BA and MC

Primary BA were obtained from the peripheral blood of 5 healthy donors and 5 patients allergic to the birch pollen allergen Bet v 1. For surface staining experiments, BA were examined in heparinized whole blood samples. For histamine release experiments, BA were enriched by dextran sedimentation [41]. MC-cultures were generated using CD133+ cord blood progenitors as reported [42]. In brief, CD133+ progenitors were isolated from cord blood mononuclear cells (MNC) using magnetic microbeads and the QuadroMACS magnetic separator (Miltenyi Biotec). The purity of isolated CD133+ cells was >97%. Isolated cells (0.5×10^6^/ml) were cultured in 6-well plates (Costar, Cambridge, MA) in SFM with SCF (100 ng/ml), IL-6 (100 ng/ml), and IL-3 (30 ng/ml) for 2 weeks, and thereafter in SCF and IL-6 without IL-3. After 4 weeks, RPMI 1640 medium containing 10% FCS was used instead of SFM. Cytokines were replaced weekly. After 7 weeks, 70–80% of cells were MC as evidenced by Wright-Giemsa staining. Human lung MC were enriched from surgical specimens of 5 patients with bronchiogenic carcinoma as described [43]. In brief, tissue was placed in Tyrode's buffer, cut into small pieces, and washed extensively in Tyrode's buffer. Then, tissue fragments were incubated in collagenase type II at 37°C for 60 minutes. Isolated cells were examined for viability and percentage of MC by Giemsa staining. Lung MC were kept in culture in RPMI 1640 medium and 10% FCS at 37°C for 2–5 days before used. In 5 patients with SM, bone marrow MNC were isolated using Ficoll.

### Culture of KU812 cells and HMC-1 cells

The human BA cell line KU812 was kindly provided by Dr. K. Kishi (Kumamoto University, Kumamoto, Japan). KU812 cells were cultured in RPMI1640 medium with 10% FCS (37°C, 5% CO_2_). The human MC leukemia cell line HMC-1 [44] was kindly provided by Dr. J. H. Butterfield (Mayo Clinic, Rochester, MN). Two subclones of HMC-1 were used: HMC-1.1 harboring the *KIT* mutation V560G, and HMC-1.2 harboring *KIT* V560G and *KIT* D816V [36]. HMC-1 cells were grown in IMDM plus 10% FCS (37°C, 5% CO_2_).

### Measurement of ^3^H-thymidine uptake

HMC-1 cells, KU812 cells, and primary bone marrow derived cells obtained from 6 patients with SM (indolent SM, n = 5, smoldering SM, n = 1) were incubated in control medium or various concentrations of NVP-BEZ235 (0.001–5 µM) at 37°C for 48 hours. Cell lines were also incubated with RAD001 (0.001–1 µM) for 48 hours. Thereafter, 0.5 µCi ^3^H-thymidine was added (37°C, 16 hours). Cells were harvested on filter membranes (Packard Bioscience, Meriden, CT) in a Filtermate 196 harvester (Packard Bioscience). Filters were air-dried, and the bound radioactivity was counted in a β-counter (Top-Count NXT, Packard Bioscience). All experiments were performed in triplicates.

### Evaluation of apoptosis by morphology and Tunel assay

The effects of NVP-BEZ235 and RAD001 on apoptosis in KU812 cells and HMC-1 cells were analyzed by morphologic examination (light microscopy) and a Tunel (in situ Terminal transferase-mediated dUTP-fluorescence Nick End-Labeling) assay using the ‘In situ cell death detection kit-fluorescein’ (Roche Diagnostics, Mannheim, Germany) [45]. For morphologic examination, cells were incubated with NVP-BEZ235 (0.001–10 µM) and RAD001 (0.001–10 µM), LY294002 (20 µM), or control medium at 37°C for 48 hours. The percentage of apoptotic cells was quantified on Wright-Giemsa-stained cytospin slides. In Tunel assay experiments, cells were incubated with NVP-BEZ235 (1 or 10 µM), RAD001 (1 or 10 µM), or control medium at 37°C for 24 hours. The Tunel assay was performed as reported [45]. Cells were analyzed on an Olympus AX-1 fluorescence microscope equipped with 100×/1.35 UPlan-Apo objective lense (Olympus). Figure acquisition was performed using Olympus DP11 camera and Adobe Photoshop CS2 software version 9.0 (Adobe Systems, San Jose, CA). Magnification, ×400.

### Application of NVP-BEZ235 in a xenotransplantation model

Female NMRI-Foxn1^nu^ mice (4–5 week old) were purchased from Charles River Laboratories (Sulzfeld, Germany). Mice were housed in barrier facilities with a 12 hour-light/dark cycles. In each mouse, 3×10^7^ HMC-1.2 cells were inoculated subcutaneously in the right and left flank. Five days after inoculation, mice were divided into two groups, a control group (n = 6 or n = 5) and a treatment group (n = 7 or n = 5). In the treatment group, mice received 40 mg/kg NVP-BEZ235 dissolved in 200 µl N-methyl-2-pyrrolidone (NMP) and polyethylene glycol 300 (PEG300; Sigma-Aldrich) or 5 mg/kg RAD001 dissolved in 200 µl distilled water *per os* (gavaging) daily for up to 22 days. In the control group, mice received NMP and PEG300 alone or sodium chloride (water control). Tumor size was inspected daily and was measured (in mm^3^) by caliper using the formula: a^2^ * b/2 (a: length of tumor; b: tumor width). On day 22, mice were sacrificed by euthanization. Tumors were collected and dispersed by collagenase digestion [44], [45]. In each case, xenotransplant tumor-derived cells were found to exhibit morphologic and phenotypic properties of HMC-1 cells (not shown).

### BA differentiation assay and MC differentiation assay

CD133+ cord blood progenitor cells were isolated from MNC using magnetic beads as reported [42]. Isolated progenitors were cultured in control medium or in the presence of either IL-3 (100 ng/ml) (BA differentiation assay) or SCF (100 ng/ml) plus IL-6 (100 ng/ml) (MC differentiation assay) at 37°C for 14 days (BA) or 28 days (MC) as reported [46], [47] Thereafter, cultures were harvested and examined for total cell counts, percentage of BA or MC by Giemsa-staining, and absolute numbers of BA and MC per well. For further objective quantification of BA- and MC differentiation, equal suspension volumes of day-14-samples (BA) or day-28-samples (MC) were examined for total cellular histamine-content by RIA as reported [46], [47].

### Staining with mAb and flow cytometry

To examine drug effects on expression of CD63 and CD203c, flow cytometry was performed. BA were incubated with NVP-BEZ235 (0.001–10 µM), RAD001 (0.001–10 µM), LY294002 (0.01–30 µM), wortmannin (0.0001–1 µM), or control medium for 15 minutes (37°C). Then, BA were washed, incubated with anti-IgE mAb E-124.2.8 (1 µg/ml) at 37°C for 15 minutes, washed, then were subjected to erythrocyte lysis, and then analyzed by multicolor flow cytometry using mAb against CD63 and CD203c as described [31], [48], [49]. Anti-IgE-induced upregulation of CD63 and CD203c was calculated from mean fluorescence intensities (MFI) obtained with stimulated (MFIstim) and unstimulated (MFIcontrol) cells, and expressed as stimulation index (SI = MFIstim∶MFIcontrol) [31], [48], [49]. Cultured IgE-loaded MC were incubated with NVP-BEZ235 (0.001–10 µM), RAD001 (0.001–10 µM), or control medium for 40 minutes (37°C). Thereafter, MC were challenged with NIP(5)-BSA (100 ng/ml) in phenolred-free medium plus 0.1% BSA for 90 minutes (37°C). Expression of CD63 and CD203c was analyzed as reported [31], [48], [49]. KU812 and HMC-1 cells were incubated with NVP-BEZ235 (0.001–10 µM), RAD001 (0.001–10 µM), LY294002 (20 µM), wortmannin (1 µM), or control medium for 24 or 48 hours, before being stained with mAb against CD63 or CD203c. Expression of cell surface antigens was analyzed on a FACSScan (Becton Dickinson Biosciences, San Jose, CA). All staining reactions were controlled by isotype-matched antibodies. For staining of cytoplasmic molecules, KU812 and HMC-1 cells were permeabilized by methanol (−20°C, 15 minutes) and then incubated with mAb against phosphorylated Akt (pAkt, S473) (M89-61), pS6 (N7-548), pSTAT5 (clone 47), or activated caspase 3 (C92-605) (all from Becton Dickinson Biosciences) for 30 minutes. Then, cells were washed and analyzed on a FACSCalibur (Becton Dickinson Biosciences). In a separate set of experiments, blood BA and lung MC were stained with mAb against CD63 and CD203c by two different staining protocols, one protocol for detection of surface expression of CD63 and CD203c (see above), and one for detection of cytoplasmic (intracellular) CD63 and CD203c. For detection of intracellular CD63 and CD203c, intact cells were first incubated with unlabeled mAb against CD63 and CD203c for 15 minutes at 4°C (to block surface antigens), washed, and then permeabilized with methanol. Thereafter, cells were stained with fluorochrome-conjugated antibodies against CD63 and CD203c (30 minutes at RT). Apoptosis was measured in drug-exposed cells by combined AnnexinV/PI staining as reported [36]. For cell cycle studies, drug-exposed cells were resuspended in 500 µL permeabilization buffer (0.1% Na-acetate and 0.1% Triton X-100). Then 40 µl PI were added, and cell cycle distribution analyzed on a FACSCalibur.

### Western blot analysis

Prior to Western blotting, HMC-1 cells and KU812 cells (each 10^6^ cells/ml) were incubated with control medium, NVP-BEZ235 (1 µM) or RAD001 (1 µM) at 37°C for 4 hours. Then, Western blotting was performed as described [36] using a polyclonal antibody against pAkt (S473) (Santa Cruz, Santa Cruz, CA), a mAb against pAkt (193H12), a polyclonal antibody against Akt (Cell Signaling, Danvers, MA), a mAb against pSTAT5 (clone 47), a mAb against total STAT5 (clone 89), a mAb against pS6 (N7-548) (all from Becton Dickinson Biosciences), and a polyclonal rabbit antibody against β-Actin (Sigma-Aldrich, St. Louis, MO). Additional Western Blot experiments were performed using polyclonal antibodies against pERK (Thr202/Tyr204), total ERK, p4EBP1 (Ser65) and pp70S6K (Thr389). All antibodies were from Cell Signaling. Antibody reactivity was made visible by sheep anti-mouse IgG or donkey anti-rabbit IgG and Lumingen PS-3 detection reagent (GE Healthcare).

### Evaluation of β-hexosaminidase release from cultured MC

After incubation with NIP(5)-BSA-specific IgE (Jw8, 10 µg/ml) and IL-4 (25 ng/ml) overnight, MC were incubated with NVP-BEZ235 (0.001–10 µM), RAD001 (0.001–10 µM), or control medium for 30 minutes (37°C). Then, cells were washed and challenged with NIP(5)-BSA (100 ng/ml) for 90 minutes (37°C). Thereafter, cell-free supernatants were collected and β-hexosaminidase release determined as reported [42].

### Histamine release experiments

Before activated, MC were incubated with human IgE (Jw8, 10 µg/ml) and IL-4 (25 ng/ml) overnight (37°C) to induce IgE-receptor expression [42]. Dextran-enriched blood BA (1×10^6^/ml) and IgE-loaded MC (1×10^5^/ml) were incubated in control medium, NVP-BEZ235 (0.001–10 µM), RAD001 (0.001–10 µM), LY294002 (0.01–30 µM), or wortmannin (0.0001–1 µM), at 37°C for 30 minutes. Thereafter, cells were exposed to anti-IgE antibody E-124.2.8 (BA: 1 µg/ml; MC: 10 µg/ml) in HRB or control buffer (HRB) at 37°C for 30 minutes. BA from allergic patients were incubated with recombinant Bet v 1 (0.1 µg/ml) (Biomay Vienna, Austria) [50] or control buffer (HRB) for 30 minutes. In select experiments, BA were challenged with Ca-ionophore A23187 (0.001–10 µg/ml) or C5a (0.01–100 nM) as reported [51]. In another set of experiments, BA or MC were incubated with control medium or NVP-BEZ235 (10 µM) for 30 minutes, and then were exposed to various concentrations of anti-IgE (0.001–10 µg/ml) for 30 minutes. After incubation, cells were centrifuged at 4°C. Cell-free supernatants and suspensions were then recovered, and samples (supernatants and cell lysates) examined for histamine content by RIA. Histamine release was calculated as percentage of released histamine compared to total (cellular+extracellular) histamine. All experiments were performed in triplicates.

### Statistical analysis

To determine the level of significance in drug inhibition experiments, standard statistical tests including the Student's t test, were applied. A p value of less than 0.05 was considered to indicate statistical significance.

## Supporting Information

Figure S1 **Effects of LY294002 and wortmannin on proliferation of HMC-1 cells and KU812 cells.** HMC-1.1 cells (left panels), HMC-1.2 cells (middle panels), and KU812 cells (right panels) were cultured in control medium (Co), control medium with DMSO control (DMSO Co), or with increasing concentrations of LY294002 (0.1 µM–20 µM) or wortmannin (0.001–1 µM) at 37°C for 48 hours. Thereafter, ^3^H-thymidine uptake was measured. Results show the percentage of ^3^H-thymidine uptake compared to control (Co) and represent the mean±S.D. of three independent experiments in each cell line. Asterisk (*): p<0.05.
(TIF)

Figure S2 **Effects of NVP-BEZ235, RAD001, LY294002 and wortmannin on cell cycle analysis of HMC-1 cells and KU812 cells.** Cell cycle distribution in HMC-1.1 cells (upper panel), HMC-1.2 cells (middle panel) and KU812 cells (lower panel) after exposure to control medium (Co) or various concentrations of NVP-BEZ235 and RAD001 (left panels) as well as LY294002 and wortmannin (right panels) as indicated, at 37°C for 48 hours. Cell cycle distribution was analyzed by flow cytometry as described in the text.
(TIF)

Figure S3 **Effects of NVP-BEZ235 and RAD001 on expression of pERK in HMC-1 cells and KU812 cell.** KU812 cells (left panel), HMC-1.2 cells (middle panel), and HMC-1.1 cells (right panel) were cultured in the absence or presence of NVP-BEZ235 (1 µM) and RAD001 (1 µM) at 37°C for 4 hours. Thereafter, cells were lysed and Western blotting was performed using antibodies against pERK and total ERK as described in the text.
(TIF)

Figure S4 **Effects of LY294002 and wortmannin on IgE-mediated histamine release in human BA.** BA obtained from healthy donors (n = 3) were preincubated with control medium (Medium Co) or various concentrations of LY294002 (left panel) and wortmannin (right panel) as indicated at 37°C for 30 minutes. Afterwards, cells were exposed to anti-IgE (1 µg/ml) at 37°C for 30 minutes. After centrifugation, histamine concentrations were determined in supernatants and cell-lysates by radioimmunoassay. Histamine release is expressed as percentage of total histamine. Results represent the mean±S.D. from three donors. Asterisk (*) indicates p<0.05.
(TIF)

Figure S5 **Effects of LY294002 and wortmannin on expression of activation-linked cell surface antigens on human BA and on expression of CD63 on KU812 cells.** (A): BA in whole blood samples were preincubated in control medium (Medium Co) or in medium containing various concentrations of LY294002 (0.01–30 µM; upper panels) or wortmannin (0.0001–1 µM; lower panels) at 37°C for 15 minutes. Then, cells were exposed to anti-IgE antibody E-124.2.8 (1 µg/ml) for another 15 minutes (37°C). Thereafter, cells were stained with monoclonal antibodies directed against CD63 (left panels) or CD203c (right panels), and analyzed by multicolor flow cytometry as described in the text. Basophils were defined as CD203c-positive cells in all samples. Anti-IgE-induced upregulation of CD antigens was calculated from mean fluorescence intensities (MFIs) obtained with stimulated (MFIstim) and unstimulated (MFIcontrol) cells and was expressed as stimulation index (SI = MFIstim∶MFIcontrol). Results show SI values and represent the mean±S.D. from three donors. Asterisk (*) indicates p<0.05 compared to Medium control. (B): KU812 cells were cultured in control medium (Co), LY294002 (20 µM), wortmannin (1 µM), or NVP-BEZ235 (0.001–10 µM) at 37°C for 24 hours (black bars) or 48 hours (open bars). After incubation, cells were stained with anti-CD63 antibody and analyzed by flow cytometry. Results show staining index (MFI corrected for the isotype control) and are expressed as mean±S.D of three independent experiments. Asterisk (*) indicates p<0.05.
(TIF)
