# Supplementary figures and images for: The PI3-Kinase/mTOR-Targeting Drug NVP-BEZ235 Inhibits Growth and IgE-Dependent Activation of Human Mast Cells and Basophils
Source: PLoS One. 2012 Jan 27;7(1):e29925. doi: 10.1371/journal.pone.0029925 (PMC3267720; doi:10.1371/journal.pone.0029925)

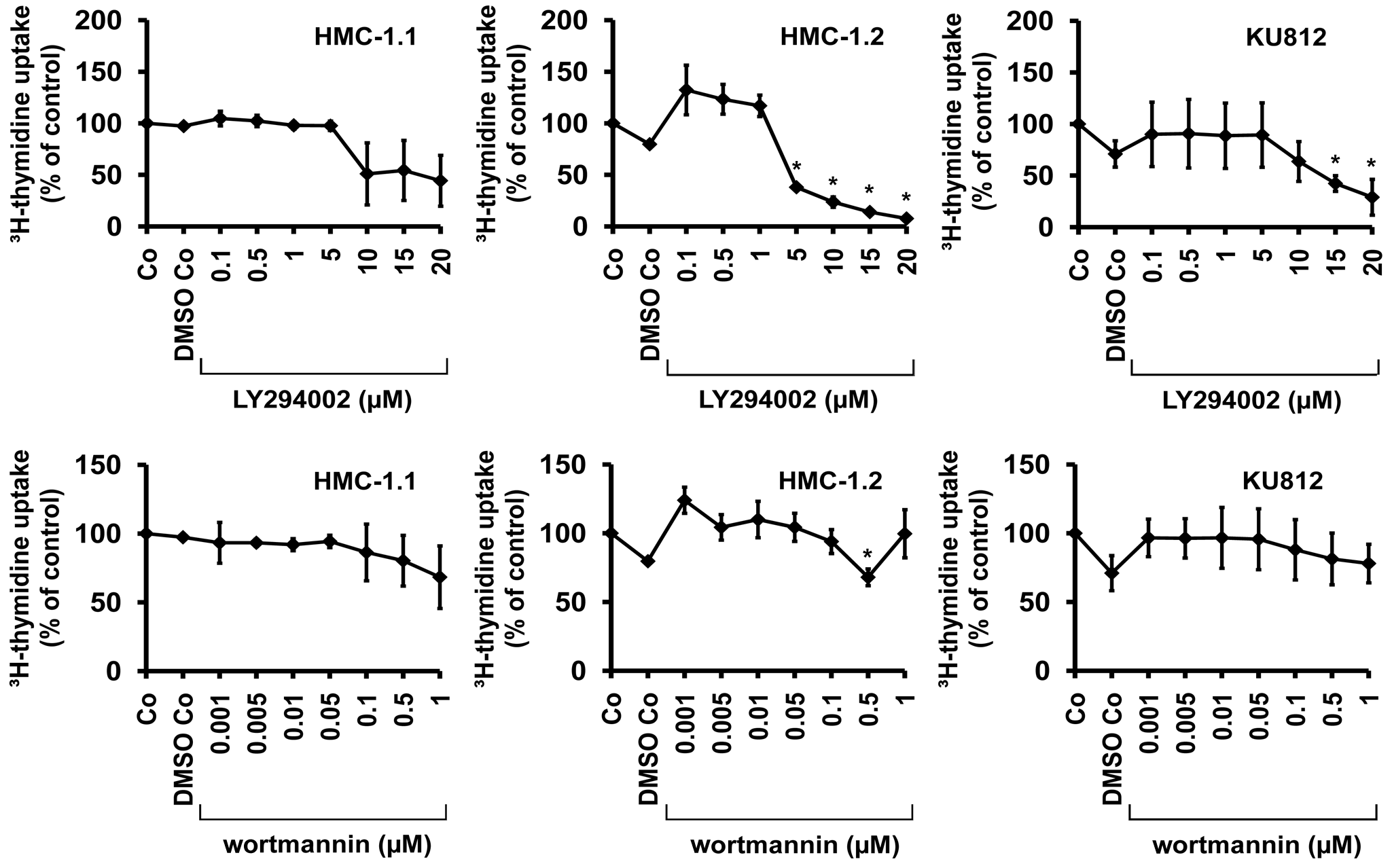

Supplement: Figure S1 — Effects of LY294002 and wortmannin on proliferation of HMC-1 cells and KU812 cells. HMC-1.1 cells (left panels), HMC-1.2 cells (middle panels), and KU812 cells (right panels) were cultured in control medium (Co), control medium with DMSO control (DMSO Co), or with increasing concentrations of LY294002 (0.1 µM–20 µM) or wortmannin (0.001–1 µM) at 37°C for 48 hours. Thereafter, 3H-thymidine uptake was measured. Results show the percentage of 3H-thymidine uptake compared to control (Co) and represent the mean±S.D. of three independent experiments in each cell line. Asterisk (*): p<0.05. (TIF) [file pone.0029925.s001.tif]

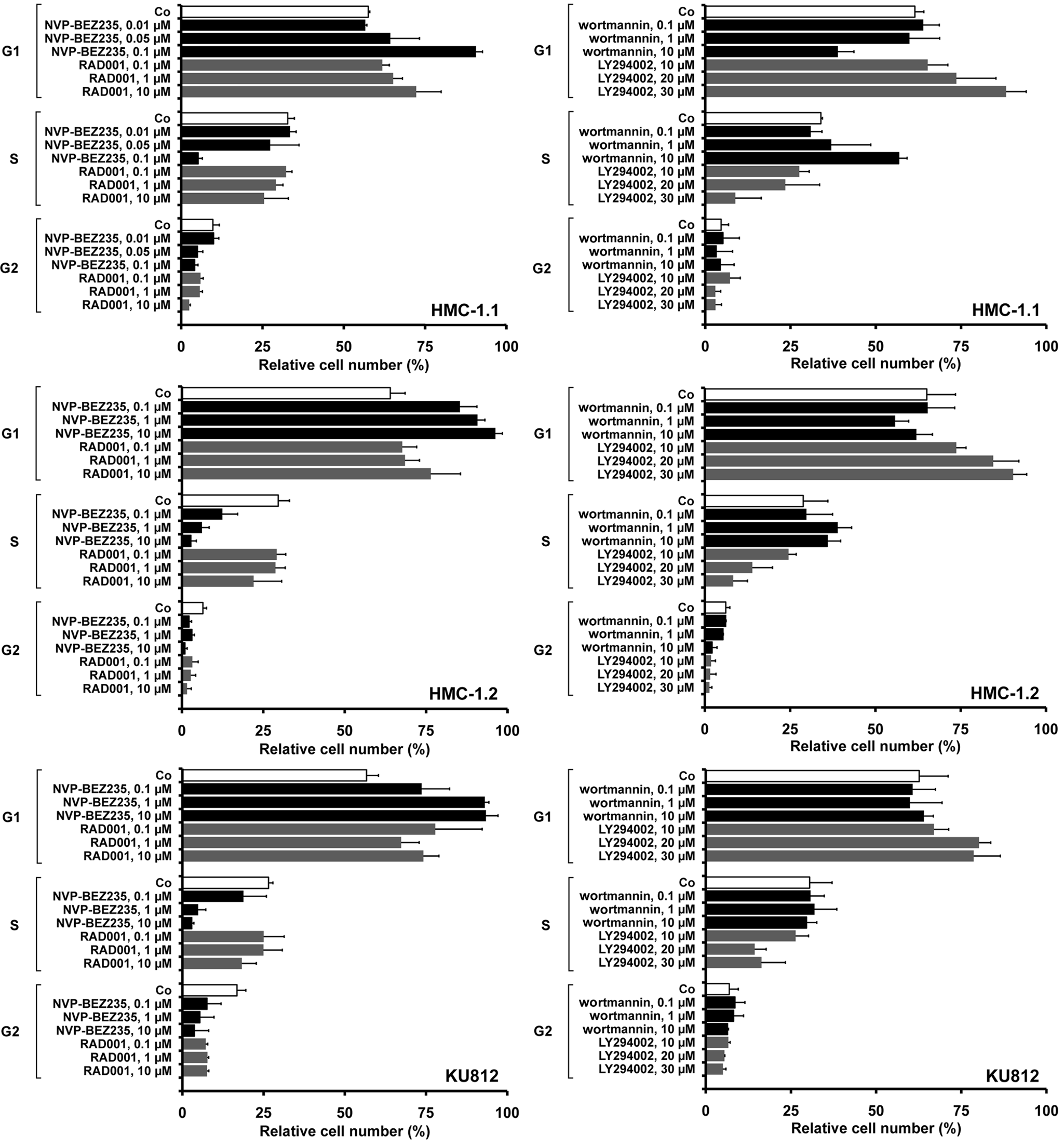

Supplement: Figure S2 — Effects of NVP-BEZ235, RAD001, LY294002 and wortmannin on cell cycle analysis of HMC-1 cells and KU812 cells. Cell cycle distribution in HMC-1.1 cells (upper panel), HMC-1.2 cells (middle panel) and KU812 cells (lower panel) after exposure to control medium (Co) or various concentrations of NVP-BEZ235 and RAD001 (left panels) as well as LY294002 and wortmannin (right panels) as indicated, at 37°C for 48 hours. Cell cycle distribution was analyzed by flow cytometry as described in the text. (TIF) [file pone.0029925.s002.tif]

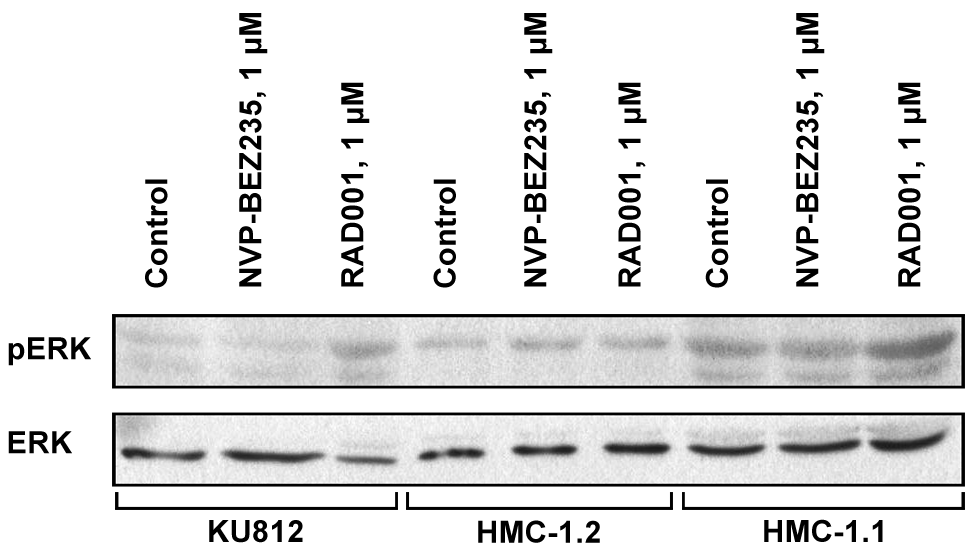

Supplement: Figure S3 — Effects of NVP-BEZ235 and RAD001 on expression of pERK in HMC-1 cells and KU812 cell. KU812 cells (left panel), HMC-1.2 cells (middle panel), and HMC-1.1 cells (right panel) were cultured in the absence or presence of NVP-BEZ235 (1 µM) and RAD001 (1 µM) at 37°C for 4 hours. Thereafter, cells were lysed and Western blotting was performed using antibodies against pERK and total ERK as described in the text. (TIF) [file pone.0029925.s003.tif]

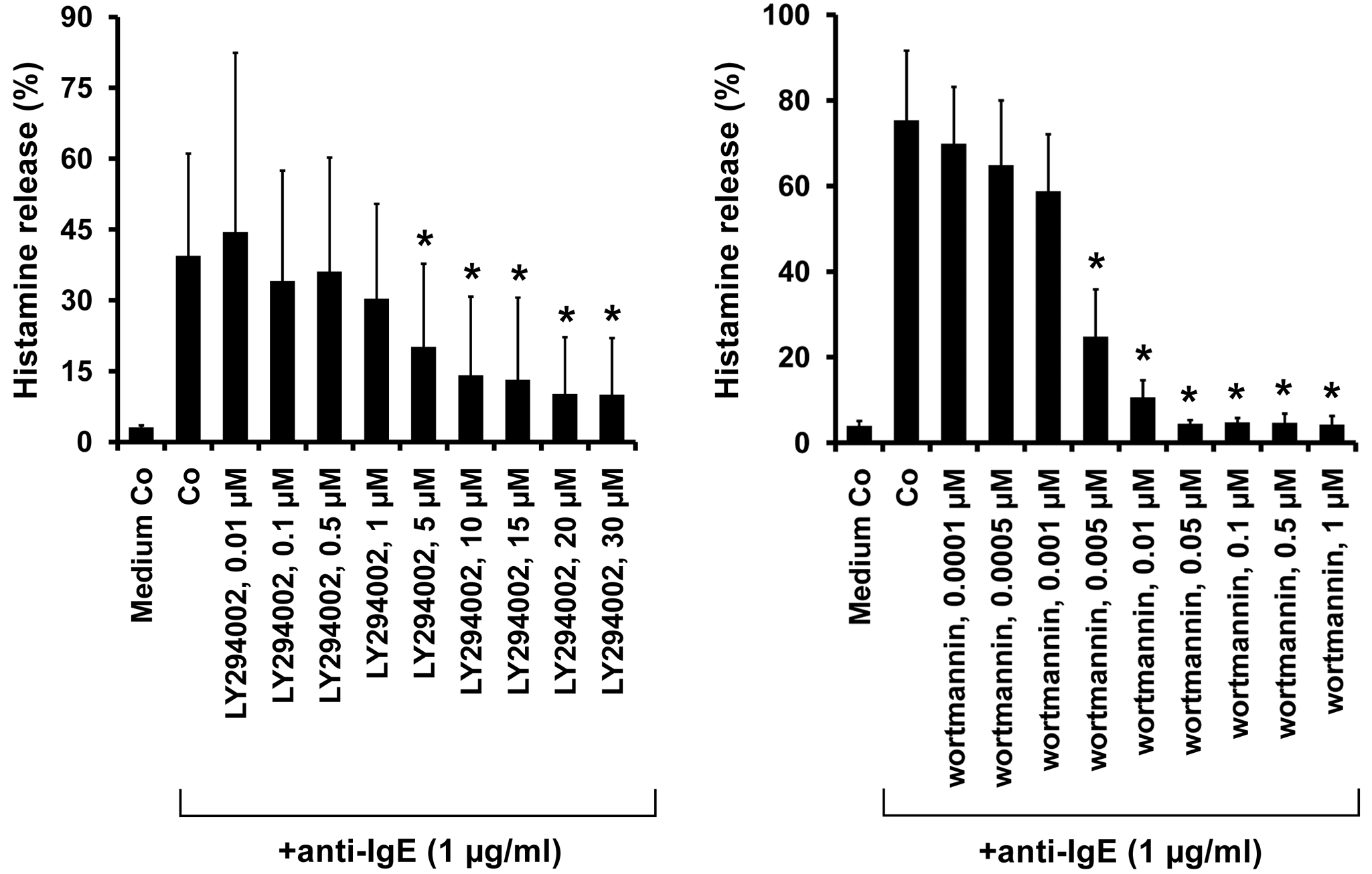

Supplement: Figure S4 — Effects of LY294002 and wortmannin on IgE-mediated histamine release in human BA. BA obtained from healthy donors (n = 3) were preincubated with control medium (Medium Co) or various concentrations of LY294002 (left panel) and wortmannin (right panel) as indicated at 37°C for 30 minutes. Afterwards, cells were exposed to anti-IgE (1 µg/ml) at 37°C for 30 minutes. After centrifugation, histamine concentrations were determined in supernatants and cell-lysates by radioimmunoassay. Histamine release is expressed as percentage of total histamine. Results represent the mean±S.D. from three donors. Asterisk (*) indicates p<0.05. (TIF) [file pone.0029925.s004.tif]

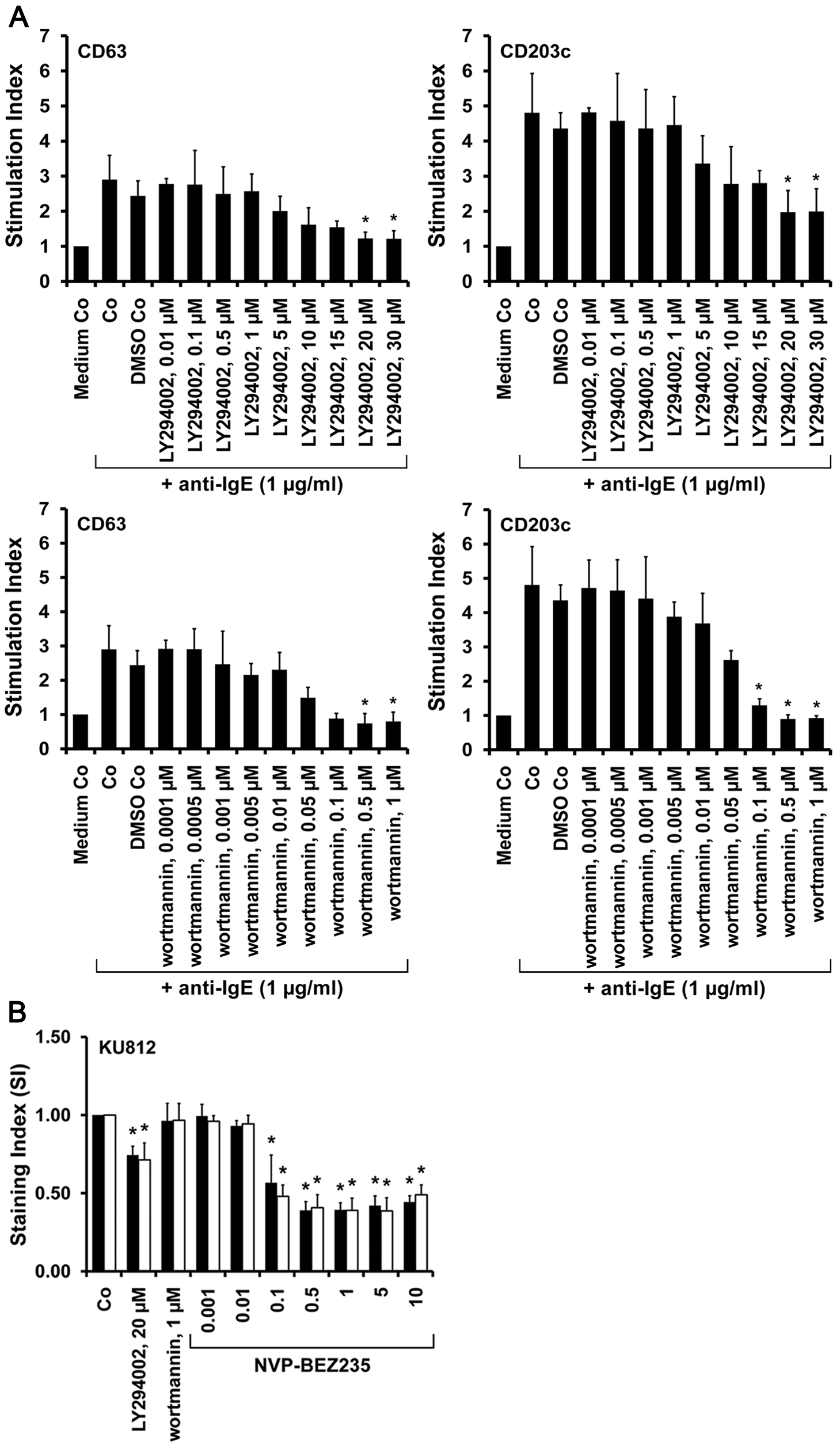

Supplement: Figure S5 — Effects of LY294002 and wortmannin on expression of activation-linked cell surface antigens on human BA and on expression of CD63 on KU812 cells. (A): BA in whole blood samples were preincubated in control medium (Medium Co) or in medium containing various concentrations of LY294002 (0.01–30 µM; upper panels) or wortmannin (0.0001–1 µM; lower panels) at 37°C for 15 minutes. Then, cells were exposed to anti-IgE antibody E-124.2.8 (1 µg/ml) for another 15 minutes (37°C). Thereafter, cells were stained with monoclonal antibodies directed against CD63 (left panels) or CD203c (right panels), and analyzed by multicolor flow cytometry as described in the text. Basophils were defined as CD203c-positive cells in all samples. Anti-IgE-induced upregulation of CD antigens was calculated from mean fluorescence intensities (MFIs) obtained with stimulated (MFIstim) and unstimulated (MFIcontrol) cells and was expressed as stimulation index (SI = MFIstim∶MFIcontrol). Results show SI values and represent the mean±S.D. from three donors. Asterisk (*) indicates p<0.05 compared to Medium control. (B): KU812 cells were cultured in control medium (Co), LY294002 (20 µM), wortmannin (1 µM), or NVP-BEZ235 (0.001–10 µM) at 37°C for 24 hours (black bars) or 48 hours (open bars). After incubation, cells were stained with anti-CD63 antibody and analyzed by flow cytometry. Results show staining index (MFI corrected for the isotype control) and are expressed as mean±S.D of three independent experiments. Asterisk (*) indicates p<0.05. (TIF) [file pone.0029925.s005.tif]
